# Establishing end-of-life boards for palliative care of patients with advanced diseases

**DOI:** 10.1007/s00508-018-1323-2

**Published:** 2018-02-23

**Authors:** Eva K. Masel, Matthias Unseld, Feroniki Adamidis, Sophie Roider-Schur, Herbert H. Watzke

**Affiliations:** 1 Clinical Division of Palliative Care, Department of Medicine I and Comprehensive Cancer Center, Medical University of Vienna, Waehringer Guertel 18-20, 1090 Vienna, Austria; 2 St. Josef Hospital, Auhofstraße 189, 1130 Vienna, Austria

**Keywords:** Neoplasms, Terminal care, Medical oncology, Palliative care, Quality of life

## Abstract

**Background:**

Interdisciplinary tumor board decisions improve the quality of oncological therapies, while no such boards exist for end-of-life (EOL) decisions. The aim of this study was to assess the willingness of hemato-oncological and palliative care professionals to develop and participate in EOL boards. An aim of an EOL board would be to establish an interdisciplinary and comprehensive care for the remaining lifetime of patients suffering from advanced incurable diseases.

**Study design:**

Staff from the interdisciplinary teams of all hemato-oncological and palliative care wards in Vienna were invited to anonymously participate in an online survey.

**Results:**

309 professionals responded. 91% respondents reported a need to establish an EOL board, 63% expressed their willingness to actively participate in an EOL board, and 25% were indecisive. Regarding patient presence, 50% voted for an EOL board in the presence of the patients, and 36% voted for an EOL board in the absence of the patients. 95% had the opinion that an EOL board could improve patient care in the last phase of life. 64% stated that the development of an EOL board would be worthwhile, while 28% did not see enough resources available at their institutions. Regarding the desired type of documentation, 61% voted for a centrally available EOL decision, and 31% supported an in-house-based documentation. 94% voted for the availability of an information folder about EOL care.

**Conclusion:**

The willingness of professionals to establish an EOL board was very high. Further steps should be taken to implement such boards to improve EOL care.

## Introduction

The last phase of life and the fact of facing death affects every individual. Medical and therapeutic treatment options for this period are increasing; however, despite all advances, treatment options go hand in hand with the necessity to inform patients about their right to decide against treatments as well as with the task to establish shared decision-making. The possibility of high-quality, individualized end-of-life (EOL) care also includes the involvement of caregivers, family, friends, society, the health system, and politics. It is evident that early palliative care and general palliative care may improve quality of life, reduce depression, and prolong life [1–5]. Regarding patients suffering from advanced cancer, numerous improvements have already been attempted and implemented in the form of interdisciplinary meetings, case conferences, psychosocial support, and tumor board decisions [6]. The implementation of such support in practice often fails due to a lack of resources [7]. Furthermore, there are still barriers regarding communication about prognosis, EOL issues, and palliative care [8, 9]. Therefore, appropriate EOL strategies are lacking in many hospitals. This study aimed to assess the willingness of hemato-oncological and palliative care professionals to develop and participate at EOL boards. The EOL boards could be offered to patients who have very limited therapeutic options, have already been heavily pretreated or have already been advised for best supportive care. For these patients, an interdisciplinary EOL board could develop a therapeutic strategy for their last phase of life and ensure the best possible preservation of their quality of life. Medical, nursing, psycho-oncological, psychosocial, and spiritual knowledge are necessary to ensure individual, professional, and sensitive care [5]. Similar to tumor board decisions, EOL board decisions should be available as documents and thus help demystify the topic of EOL. The aim of the present study was to evaluate the need to establish an EOL board and, if necessary, to put this concept into practice as a next step. For this reason, the multi-professional staff of all hemato-oncological and palliative care departments of Vienna were invited to participate in an online survey about the establishment of an EOL board. Medical doctors, nursing staff, dieticians, social workers, physiotherapists, psychologists, psychiatrists, psychotherapists, and chaplains were asked to participate.

## Methods

### Setting

Professional staff from the multidisciplinary teams of all hemato-oncological and palliative care departments in Vienna were recruited to participate in an online survey comprising 10 questions (www.surveymonkey.com). Questions related to the following topics were raised:

1. Which professional group the respondent belonged to,
2. Their attitude towards establishing an EOL board,
3. Their assessment of the need for an EOL board at their own workplace,
4. Their perception of caring for patients with incurable diseases at their workplace,
5. Their willingness to actively participate in an EOL board,
6. Their attitude towards the methods of establishing an EOL board (in the presence of patients or in the absence of patients),
7. The benefit of establishing an EOL board in the last phase of life,
8. The possibility of practically implementing an EOL board at their workplace,
9. Their desired type of documentation on an EOL board decision,
10. Their attitude towards delivering an information folder about EOL care.

According to information from the Ethics Committee of the Medical University of Vienna, an ethics approval was not necessary because of the study method (i. e. an online survey solely interviewing professionals). The online survey took place from 1 March 2016 to 31 October 2016 in two runs sent out by email (March 2016 and July 2016). Before, the survey was sent to the medical as well as to the nursing management of the following institutions for approval: General Hospital Vienna, Hospital Barmherzige Brueder, Caritas Socialis Hospice Rennweg, Kaiser Franz Josef Hospital, Hospital Goettlicher Heiland, Hospital St. Elisabeth, St. Josef Hospital, Hanusch Hospital, Hospital Hietzing, Hospital Rudolfstiftung, Hospital Wilhelminenspital and SMZ Ost Hospital. The study was approved at all institutions. The survey was conducted in German.

### Statistical analysis

The data were analyzed using descriptive statistics.

## Results

**Fig. 1:**
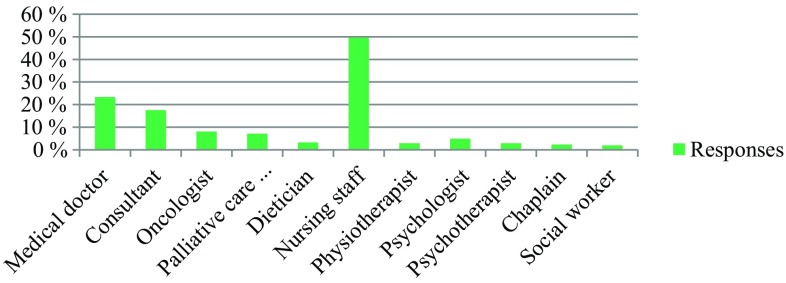
Question 1: Which professional group do you belong to? (multiple answers possible)

**Fig. 2:**
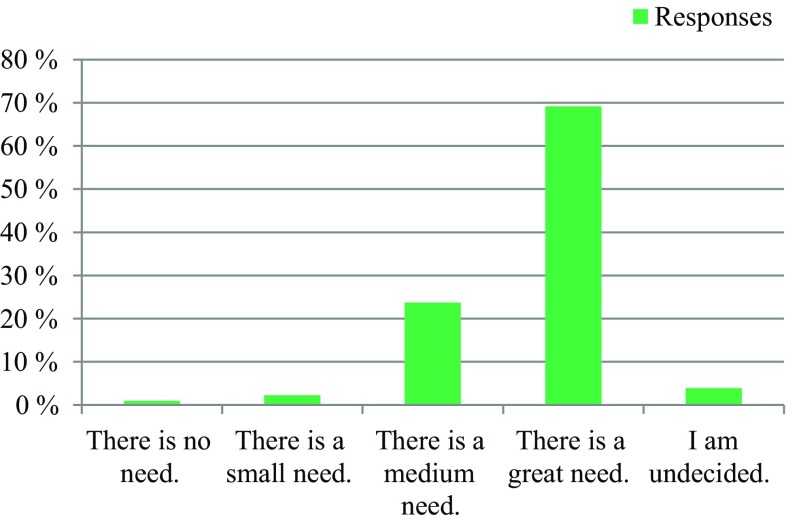
Question 2: How do you rate the need to establish an end of life board?

**Fig. 3:**
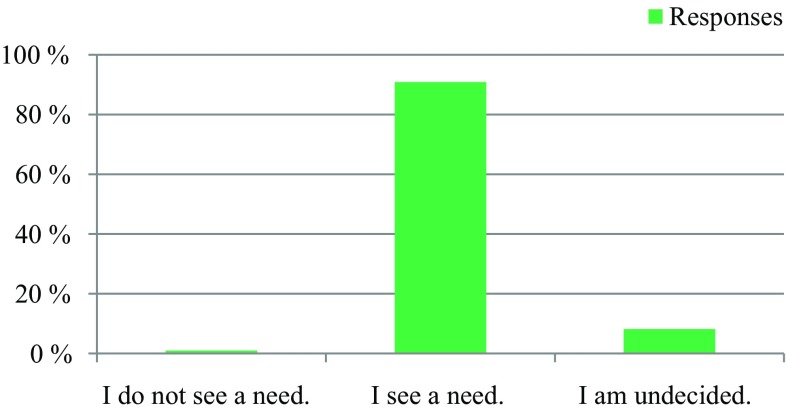
Question 3: What do you think about establishing an end of life board for patients with a limited life expectancy of less than 2 years?

**Fig. 4:**
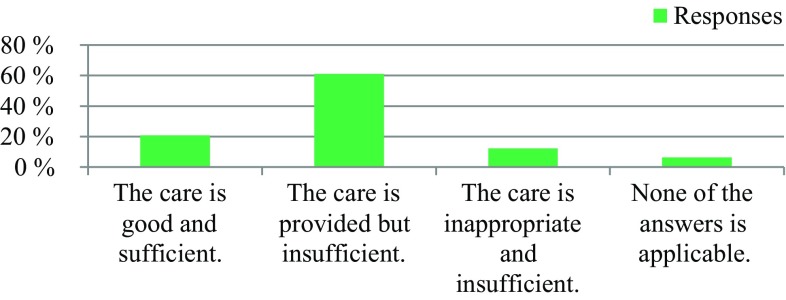
Question 4: How do you perceive the care of patients with incurable diseases at your personal workplace?

**Fig. 5:**
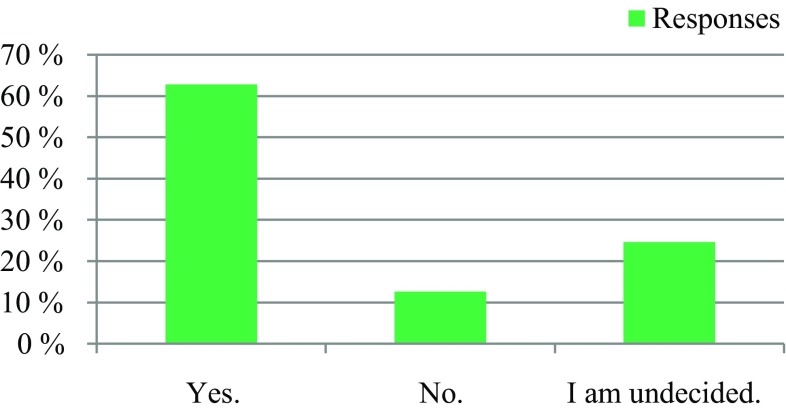
Question 5: Would you be willing to actively participate in an end of life board?

**Fig. 6:**
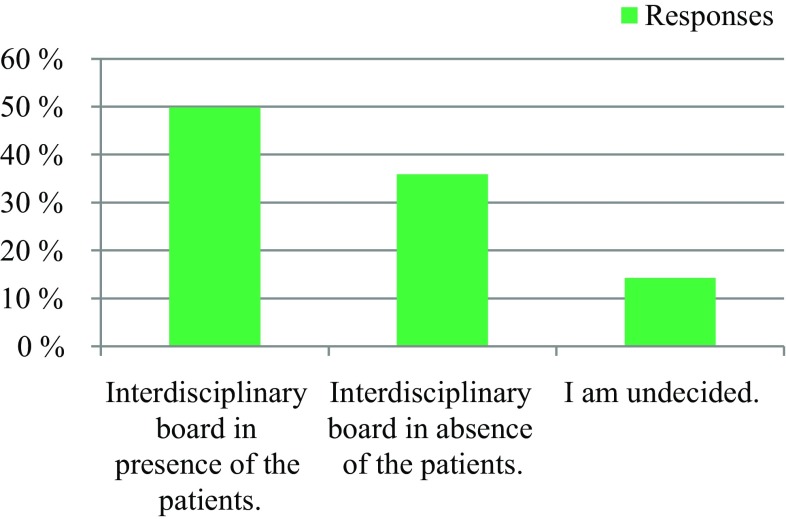
Question 6: Which form of an end of life board would you prefer?

**Fig. 7:**
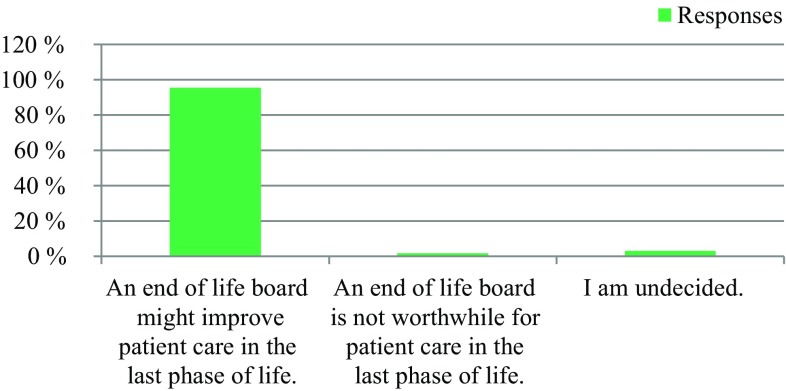
Question 7: How do you perceive the establishment of an end of life board in terms of patient care in the last phase of life?

**Fig. 8:**
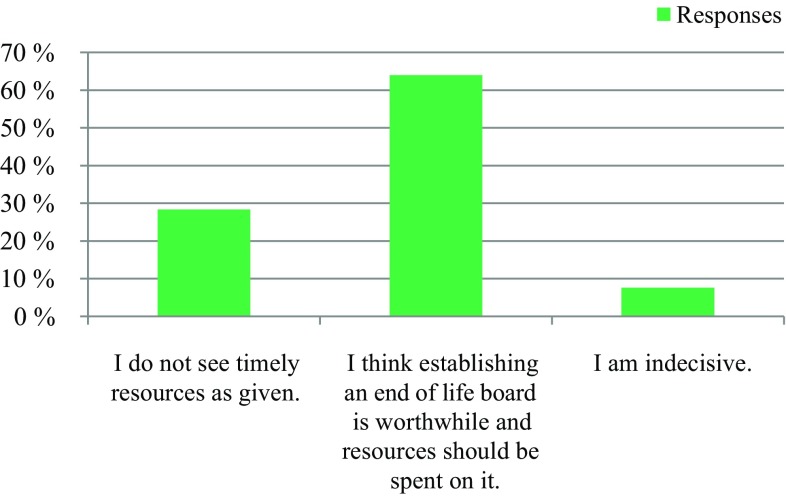
Question 8: How do you consider a practical implementation of an End of Life Board at your institution?

**Fig. 9:**
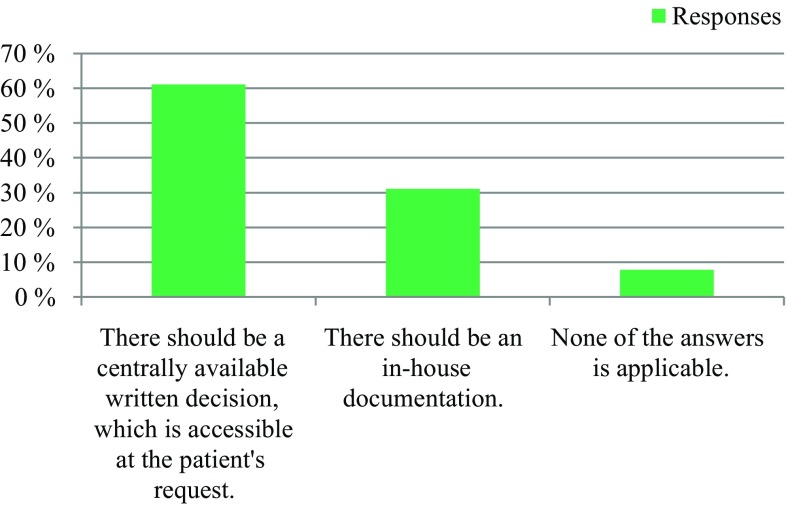
Question 9: How do you think documentation of an end of life board decision should be?

**Fig. 10:**
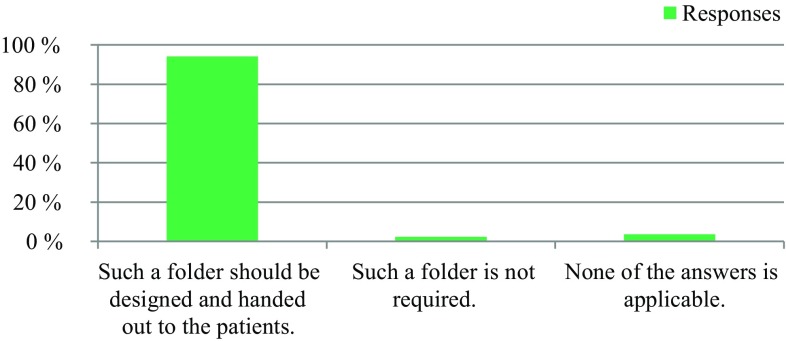
Question 10: What is your opinion towards handing out an information folder with information concerning the last phase of life (hospice, palliative care, mobile care services, advance care planning)?

The survey was conducted anonymously. To ensure adequate data protection, it was not possible to identify the participants. In the questionnaire itself, no email addresses or names of people or institutions were requested. All responses were made anonymously via the questionnaire server www.surveymonkey.com. Double responses were prevented by the online system. There were 309 people who participated in the survey. The results are presented in Fig. 1, 2, 3, 4, 5, 6, 7, 8, 9 and 10. The responding professional groups (multiple answers were possible) were 49.68% nursing staff, 23.38% medical staff, (17.53% consultants, 8.12% oncologists, and 7.14% palliative care specialists), 4.87% psychologists, 3.25% dieticians, 2.92% physiotherapists, 2.92% psychotherapists, 2.27% chaplains, and 1.95% social workers. Of the respondents, 90.85% saw a need to establish an EOL board for patients with a life expectancy of less than 2 years, 8.17% were indecisive, and 0.98% saw no need. With respect to the perceived need, 69.16% saw a great need to establish an EOL board, 23.70% saw a moderate need, 3.90% were indecisive, 2.27% saw a small need, and 0.97% saw no need. In addition, 61.06% perceived the care of patients suffering from incurable diseases at their workplace as provided but insufficient, 20.79% reported the care as good and sufficient, 12.21% described it as inappropriate and insufficient and for 6.27%, none of the answers were applicable. Respondents willing to actively participate on an EOL board accounted for 62.78% of the sample, while 24.60% of respondents were indecisive, and 12.62% were not willing to participate at an EOL board. Nearly half (49.83%) of the respondents voted for an EOL board in the presence of patients, 35.88% of respondents voted for an EOL board in the absence of patients, and 14.29% were indecisive. In addition, 95.44% of respondents believed that EOL boards could improve patient care in the last phase of life, 2.93% of respondents were indecisive, and 1.63% of the respondents reported that the establishment of EOL boards would not be worthwhile for the last phase of life. Nearly two thirds (64.03%) of respondents indicated that a practical implementation of an EOL board at their own institution would be worthwhile, and resources should be spent on it, 28.38% of respondents did not consider there to be enough resources to implement an EOL board and 7.59% were indecisive. With respect to the desired method of documenting an EOL board decision, 61.11% of the respondents voted for a centrally available written decision that should be made available at the patient’s request, 31.05% of respondents favored an in-house documentation and for 7.84%, none of the answers were applicable. Regarding their opinion towards handing out an information folder with facts concerning the last phase of life (e.g., hospice, palliative care, mobile care services, advance care planning), 94.14% of respondents indicated that such a folder should be designed and handed out to the patients, 3.58% of respondents stated that none of the answers were applicable, and 2.28% reported that such a folder is not required.

## Discussion

The results of the study show that the willingness of multidisciplinary professionals working in hemato-oncologic and palliative care wards to establish an EOL board is very high. The main respondents were nursing staff and medical doctors. A high percentage of the interviewees were willing to actively participate in an EOL board. Despite the fact that daily medical work is often characterized by bureaucracy and a lack of resources, professionals are very willing to participate in an EOL board according to the results of the present study. The fact that 60% of the participants considered the current provision of care for patients suffering from advanced diseases to be insufficient is a clear argument for further steps towards establishing better EOL care. This underlines the fact that the EOL phase represents an important area in hemato-oncology, and that professionals aim to improve the care of their patients and are willing to spend resources for this purpose. Since dealing with EOL issues will continue to be a taboo subject, active education might be necessary. Handing over an information folder with material concerning the last phase of life and advance directives, financial and social aspects, home care, hospices, palliative care, and mobile care service was considered to be useful by a high percentage of respondents. Thus, the results of this study will serve as a practical, relevant basis to encourage the implementation of EOL boards. One of the core tasks of palliative care is the bundling of multidisciplinary expertise. This comprehensive care becomes eminently important in the last phase of life, where personal and spiritual needs as well as financial and psychosocial aspects are relevant [10]. An EOL board involving multidisciplinary professionals should clarify if there is a possibility for home care, if one should arrange a mobile palliative care team, what kind of financial support is possible, what are the options for organizing home care, what kind of psychological care is available, what kind of spiritual accompaniment is possible, what types of advance care planning exists and what are the options for physiotherapy, for dietary counseling and for evidence-based complementary medical procedures. These questions could be summarized in an EOL decision, which could result in a centrally accessible written document made available at the patient’s request.

In addition, the use of an EOL information folder was favored by the participants. This folder could be created and handed out so that patients and their caregivers receive bundled information that they are often missing. Previous studies have shown that confronting patients with their prognosis does not affect the well-being of patients, and that the majority of patients wish to be informed [11]. Nevertheless, an active approach to the patients concerning EOL topics is still not common medical practice [12–14]. A high percentage of patients in Austria die in hospitals, while a very small proportion of Austrians, <4%, have advance directives. Ongoing studies as well as experience from palliative care teams show that a high proportion of patients suffering from advanced diseases want to make provisions for the last stage of life, but there is a lack of information on advance care planning [15].

Another aim of this study was to question the preference as to whether EOL boards should take place in the presence or absence of the patients. The results showed that half of the respondents favored the presence of the patients, while almost a quarter opposed the presence of the patients. Both variants could be tested in a pilot test.

The limitations and strengths of the current study should be mentioned. A limitation of this study was that the exact number of professionals to whom the questionnaire was sent as well as the drop-out rate could not be detected. Therefore, the reasons why participants were unwilling to take part in the survey could not be determined. Hence, there was a possible selection bias towards participants who found the subject area of the survey important and tended towards positive answers. Thus, critical opinions could have been obscured. Furthermore, some professional groups in the multidisciplinary team were underrepresented, which, however, corresponds to everyday work at palliative care wards. The strengths of this study include the high response rate, which was achieved by using a web-based online questionnaire that took little time to complete. Thus, the response rate was satisfactory. Another strength was the novelty of the research question.

## Conclusion

The willingness to establish an EOL board was very high among hemato-oncological staff, with 91% of respondents supporting this idea. A high number of participants (94%) voted to hand out an information folder with material regarding the last phase of life. Data from the current study found that respondents saw a need to establish an EOL board, that they would be willing to spend resources on this topic, and that most would actively participate. Thus, the results of the current study should serve as a basis to underline the importance of establishing EOL boards. Referring to these study results and to the fact that tumor boards are already established at Austrian oncology centers, the establishment of EOL boards at comprehensive cancer centers could help to improve the care of patients suffering from advanced diseases.
